# Supplementary material for: Apoptosis recognition receptors regulate skin tissue repair in mice
Source: eLife. 2023 Dec 21;12:e86269. doi: 10.7554/eLife.86269 (PMC10735221; doi:10.7554/eLife.86269)
Supplement: Supplementary file 1. [file elife-86269-supp1.docx]

| Sample ID | Diabetic (Y/N) | Age at time of collection (years) | Sex (F/M) | Race | Sample type |
| --- | --- | --- | --- | --- | --- |
| Diac32 | Y | 51 | M |  | scRNA-seq |
| Diac34 | Y | 34 | M |  | scRNA-seq |
| Diac35 | Y | 54 | M |  | scRNA-seq |
| Diac36 | Y | 68 | M |  | scRNA-seq |
| Diac37 | Y | 81 | M |  | scRNA-seq |
| Diac38 | N | 66 | M |  | scRNA-seq |
| Diac39 | N | F | 85 |  | scRNA-seq |
| Diac44a | N | M | 55 | Black | scRNA-seq |
| Diac44b | Same subject as above |  |  |  | scRNA-seq |
| Diac44c | Same subject as above |  |  |  | scRNA-seq |
| Diac50a | N | F | 66 |  | scRNA-seq |
| Diac50b | Same subject as above | -- | -- |  | scRNA-seq |
| NDFW D47 | N |  | M |  | IF |
| NDFW D48 | N |  | M |  | IF |
| DFU D58G | Y |  |  |  | IF |
| DFU D59A | Y |  | M |  | IF |
| DFU D64A | Y |  | M | White | IF |
| DFU 68G | Y |  | M |  | IF |
| DFU 72A | Y |  | M |  | IF |
| DFU D80A | Y (T1) |  | F | White | IF |
